# Pterostilbene Induces Cell Apoptosis and Cell Cycle Arrest in T-Cell Leukemia/Lymphoma by Suppressing the ERK1/2 Pathway

**DOI:** 10.1155/2017/9872073

**Published:** 2017-07-12

**Authors:** Gaomei Chang, Wenqin Xiao, Zhijian Xu, Dandan Yu, Bo Li, Yong Zhang, Xi Sun, Yongsheng Xie, Shuaikang Chang, Lu Gao, Gege Chen, Liangning Hu, Bingqian Xie, Bojie Dai, Weiliang Zhu, Jumei Shi

**Affiliations:** ^1^Department of Hematology, Anhui Medical University, Hefei 230032, China; ^2^Department of Hematology, Shanghai Tenth People's Hospital, Tongji University School of Medicine, Shanghai 200072, China; ^3^CAS Key Laboratory of Receptor Research, Drug Discovery and Design Center, Shanghai Institute of Materia Medica, Chinese Academy of Sciences, Shanghai 201203, China; ^4^State Key Laboratory of Medicinal Chemical Biology, Nankai University, Tianjin 300071, China; ^5^College of Life Science and Technology, Tongji University, Shanghai 200092, China

## Abstract

Pterostilbene is a natural 3,5-dimethoxy analog of* trans*-resveratrol that has been reported to have antitumor, antioxidant, and anti-inflammatory effects. T-cell leukemia/lymphoma is one of the more aggressive yet uncommon non-Hodgkin lymphomas. Although there has been increasing research into T-cell leukemia/lymphoma, the molecular mechanisms of the antitumor effects of pterostilbene against this malignancy are still largely unknown. The aim of this study is to confirm the effects of pterostilbene in T-cell leukemia/lymphoma. Jurkat and Hut-78 cells treated with pterostilbene were evaluated for cell proliferation using Cell Counting Kit-8, and apoptosis, cell cycle progression, reactive oxygen species generation, and mitochondrial membrane potential were analyzed using flow cytometry. The level of protein expression was detected by western blot. The results demonstrated that pterostilbene significantly inhibited the growth of T-cell leukemia/lymphoma cell lines in vitro and induced apoptosis in a dose- and time-dependent manner. Moreover, pterostilbene treatment markedly induced S-phase cell cycle arrest, which was accompanied by downregulation of cdc25A, cyclin A2, and CDK2. Pterostilbene also induced the generation of reactive oxygen species and the loss of mitochondrial membrane potential and inhibited ERK1/2 phosphorylation. Taken together, our study demonstrated the potential of pterostilbene to be an effective treatment for T-cell leukemia/lymphoma.

## 1. Introduction

T-cell leukemia/lymphoma was grouped into 22 subtypes by the 2008 World Health Organization classification system and is one of the more aggressive and uncommon non-Hodgkin lymphomas [1]. The incidence of T-cell leukemia/lymphoma accounts for 30% of all non-Hodgkin lymphomas in Asia [2]. T-cell leukemia/lymphoma has a poor prognosis and is more aggressive than B-cell lymphoma. Traditional therapy does not provide satisfactory results, and most patients relapse after primary therapy. Although there has been increasing research into T-cell leukemia/lymphoma, the molecular mechanism of pterostilbene against this malignancy, as well as its efficacy in clinical application, remains largely unknown. Therefore, studies such as this are essential to develop novel chemopreventive and/or chemotherapeutic agents to improve treatment for human T-cell leukemia/lymphoma.

Resveratrol, the most investigated polyphenol, is a promising chemopreventive agent for cancer treatment. Resveratrol is considered an effective antitumor agent due to its low toxicity and ability to regulate complicated cancer-associated molecular pathways [3]. Jang et al. demonstrated that resveratrol has anticarcinogenic effects in a two-stage murine skin cancer model [4]. Pterostilbene, a resveratrol-like agent, is an antitumor compound found in grapes and other foods [5]. Several studies have indicated that pterostilbene possesses higher antifungal effects and has greater bioavailability than resveratrol [6, 7]. Moreover, pterostilbene also has stronger lipophilicity and is easier to absorb than resveratrol. Studies have shown that pterostilbene induces cancer cell apoptosis in breast, liver, and lung cancers [8–10]. Pterostilbene also has other pharmacologic properties, including anti-inflammatory, antioxidant, and analgesic activity [11]. The mitogen-activated protein kinase (MAPK) pathways are known to play roles in protumor processes, such as promoting proliferation and blocking apoptosis. It has been confirmed in the hematopoietic system that pterostilbene has an antitumor activity against multiple myeloma and B-cell lymphomas, via suppressing MAPK signaling [12, 13].

In this study, we show that pterostilbene inhibits proliferation and induces apoptosis and cell cycle arrest in T-cell leukemia/lymphoma cells. In addition, we investigated the main mechanisms of pterostilbene's antitumor activity in T-cell leukemia/lymphoma cells.

## 2. Materials and Methods

### 2.1. Cell Culture

Jurkat cell line (originally called JM) was established in the late 1970s from the peripheral blood of a 14-year-old boy with T-cell leukemia [14]. Hut-78 is a cell line of cutaneous T-cell lymphoma originally derived from peripheral blood of patients with Sézary syndrome [15]. Jurkat and Hut-78 cells were purchased from American Type Culture Collection (Manassas, VA, USA) and grown in suspension in RPMI-1640 medium (Gibco, Carlsbad, CA, USA) supplemented with 10% fetal bovine serum (Gibco) and 1% penicillin-streptomycin-glutamine (Gibco). Normal peripheral blood mononuclear cells (PBMCs) were isolated from the human peripheral blood using Lymphoprep. CD34+ cells were isolated from human peripheral stem cell collection products using CD34 Progenitor Cell Isolation Kit (Miltenyi Biotec Inc., CA, USA). CD34+ cells from peripheral stem cells and PBMCs were cultured in RPMI-1640 medium containing 10% FBS. Cells were incubated at 37°C in 5% carbon dioxide.

### 2.2. Reagents

Pterostilbene (100 mM) was dissolved in dimethylsulfoxide (Sigma-Aldrich, St. Louis, MO, USA) and stored at −20°C. Anti-GAPDH, ERK1/2, p-ERK1/2, cdc25A, CDK2, cleaved caspase-3, cleaved caspase-8, caspase-9, and PARP antibodies (for western blot analysis) were purchased from Cell Signaling Technology (Danvers, MA, USA). Anti-cyclin A2 antibody was obtained from Epitomic (Burlingame, CA, USA). Cell Counting Kit-8 (CCK8) was purchased from Yeasen (Shanghai, China). JC-1 Mitochondrial Membrane Potential Detection Kit was purchased from Beyotime Institute of Biotechnology (Shanghai, China). The Cell Apoptosis Kit and propidium iodide (PI) were purchased from BD Biosciences (San Jose, CA, USA). SCH772984 (ERK inhibitor) was purchased from Target Mol (USA).

### 2.3. Cytotoxicity Assay

Jurkat and Hut-78 cells (2 × 10^5^ cells/mL) were plated onto 96-well plates and treated with different concentrations (0, 10, 15, 20, 25, 30, 35, and 40 *μ*M) of pterostilbene for 24 or 48 h and different concentrations (0, 1, 2, 5, 10, 15, and 20 *μ*M) of SCH772984 for 48 h. PBMCs were treated with different concentrations (0, 10, 20, 40, and 80 *μ*M) of pterostilbene for 48 h. The CCK-8 was used to evaluate cell proliferation, with absorbance measured at 450 nm.

### 2.4. Cell Cycle Analysis

Jurkat and Hut-78 cells (3 × 10^5^ cells/mL) were plated onto 24-well plates and treated with various concentrations (0, 5, 10, and 20 *μ*M) of pterostilbene and SCH772984 (0, 10 *μ*M) for 24 h. Then, cells were washed with ice-cold phosphate-buffered saline (PBS) and fixed with 70% ethanol at −20°C overnight. After fixing, cells were washed with PBS, stained with PI for 15 min, and analyzed on a BD FACSCanto II flow cytometer (BD Biosciences).

### 2.5. Apoptosis Analysis

Jurkat and Hut-78 cells (3 × 10^5^ cells/mL) were plated onto 24-well plates with pterostilbene (0, 20, 40, and 80 *μ*M) and incubated for 24 or 48 h and SCH772984 (0, 10 *μ*M) for 48 h. PBMCs and CD34+ cells from peripheral stem cells were treated with pterostilbene (0, 20, 40, and 80 *μ*M) and incubated for 48 h. Then, cells were stained with Annexin V-FITC/PI and detected by flow cytometry. Annexin V^+^/PI^−^-stained cells (early apoptosis) or Annexin V^+^/PI^+^-stained cells (late apoptosis) were considered apoptotic.

### 2.6. Mitochondrial Membrane Potential Analysis

Jurkat and Hut-78 cells were treated with pterostilbene (0, 20, 40, and 80 *μ*M) for 48 h. After collecting the cells, mitochondrial depolarization was evaluated by flow cytometry, using JC-1 staining, according to the manufacturer's protocol.

### 2.7. Measurement of Reactive Oxygen Species Generation

Jurkat and Hut-78 cells were treated with 10 *μ*M pterostilbene for 48 h at 37°C. After treatment, cells were resuspended in RPMI-1640 medium containing 10 *μ*M 20,70-dichlorofluorescin diacetate (DCFH-DA; Sigma-Aldrich) and incubated at 37°C for 25 min. In the negative group, Jurkat and Hut-78 cells were treated without pterostilbene and DCFH-DA. Fluorescence intensity was detected by flow cytometry.

### 2.8. Western Blot Analysis

Jurkat and Hut-78 cells were treated with different concentrations (0, 20, and 40 *μ*M and 0, 5, and 10 *μ*M, resp.) of pterostilbene and SCH772984 (10 *μ*M), and then proteins were extracted in radioimmunoprecipitation assay (RIPA) buffer (Sigma-Aldrich). Equivalent amounts of protein (30 *μ*g) were separated by 8%–12% sodium dodecyl sulfate-polyacrylamide gel electrophoresis and transferred onto polyvinyl difluoride membranes. After blocking in 5% low fat milk for 1 h, membranes were probed with primary antibodies overnight at 4°C. Membranes were washed with PBST (PBS + 0.1% Tween-20) three times and then probed with a secondary antibody for 1 h at room temperature. The intensities of immunoreactive bands were measured using an Odyssey two-color infrared laser imaging system (LI-COR, Lincoln, NE, USA).

### 2.9. Statistical Analysis

All data were reported as mean ± standard deviation. Student's two-tailed* t*-test was used to calculate the statistical significance of differences between two groups. The differences of multiple groups were calculated by one-way ANOVA with post hoc test. *P* < 0.05 was considered statistically significant.

## 3. Results

### 3.1. Pterostilbene and SCH772984 Inhibit the Growth of T-Cell Leukemia/Lymphoma Cells

**Figure 1:**
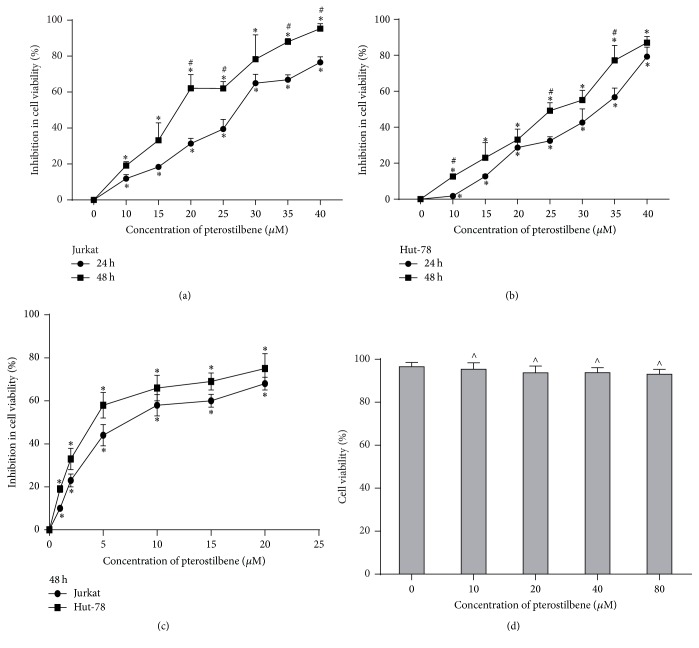
Pterostilbene and SCH772984 inhibit the growth of T-cell leukemia/lymphoma cells. (a, b) Jurkat and Hut-78 cells (2 × 10^5^ cells/mL) were plated onto 96-well plates and treated with different concentrations (0, 10, 15, 20, 25, 30, 35, and 40 *μ*M) of pterostilbene for 24 or 48 h. Data were represented as mean ± SD, *n* = 3. ^*∗*^*P* < 0.05, compared to the control group. ^#^*P* < 0.05, compared to the 24 h group. (c) Jurkat and Hut-78 cells were treated with SCH772984 (0, 1, 2, 5, 10, and 20 *μ*M) for 48 h. Data were represented as mean ± SD, *n* = 3. ^*∗*^*P* < 0.05, compared to the control group. (d) Peripheral blood mononuclear cells (PBMCs) were treated with 0, 10, 20, 40, and 80 *μ*M of pterostilbene for 48 h. Data were represented as mean ± SD, *n* = 3. ^∧^*P* > 0.05, compared to the control group. The CCK-8 was used to evaluate these cells' proliferation, with absorbance measured at 450 nm.

Jurkat and Hut-78 cells (2 × 10^5^ cells/mL) were plated onto 96-well plates and treated with different concentrations (0, 10, 15, 20, 25, 30, 35, and 40 *μ*M) of pterostilbene for 24 or 48 h and different concentrations (0, 1, 2, 5, 10, 15, and 20 *μ*M) of SCH772984 for 48 h. PBMCs were treated with 0, 10, 20, 40, and 80 *μ*M of pterostilbene for 48 h. The CCK-8 was used to evaluate cell proliferation, with absorbance measured at 450 nm. At 48 h, the calculated IC50 (50% cell growth inhibitory concentration) values were 17.83 *μ*M (Jurkat) and 22.74 *μ*M (Hut-78) with pterostilbene treatment and 8.14 *μ*M (Jurkat) and 4.81 *μ*M (Hut-78) with SCH772984 treatment. As shown in Figures 1(a) and 1(b), pterostilbene inhibited the growth of Jurkat and Hut-78 cells in a dose- and time-dependent manner. To detect the role of ERK1/2 pathway in pterostilbene treatment, we used SCH772984, a novel and selective inhibitor of ERK1/2, to perform our experiment. A recent study reported that SCH772984 inhibited the proliferation of RAS, or BRAF, mutant cancer cells [16]. Consistent with the antiproliferation effect of pterostilbene, we found that SCH772984 can inhibit the growth of T-cell leukemia/lymphoma cells (Figure 1(c)). In addition, pterostilbene has no toxicity in PBMCs (Figure 1(d)), suggesting that pterostilbene is a safe compound in our study.

### 3.2. Pterostilbene and SCH772984 Induce Cell Cycle Arrest in T-Cell Leukemia/Lymphoma Cells

**Figure 2:**
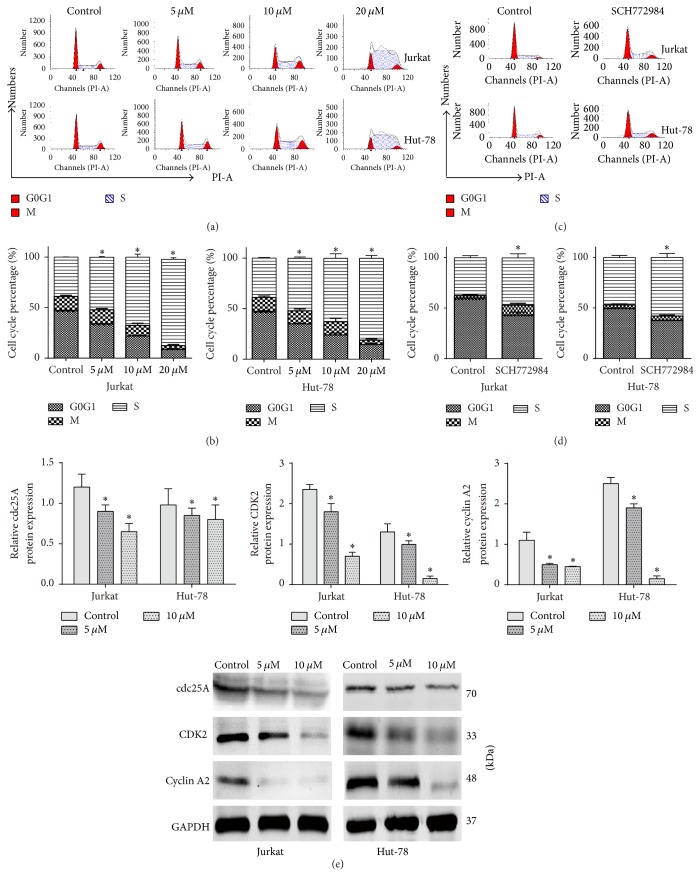
Pterostilbene and SCH772984 induce cell cycle arrest in T-cell leukemia/lymphoma cells. (a, c) Jurkat and Hut-78 cells were arrested in the S-phase treated with different concentrations of pterostilbene (0, 5, 10, and 20 *μ*M) and SCH772984 10 *μ*M for 24 h. (b, d) The percentage of G0/G1, G2M, and S-phase was indicated following various concentrations of pterostilbene and SCH772984 treatments. Data were represented as mean ± SD, *n* = 3. ^*∗*^*P* < 0.05, compared to the control group. (e) The protein levels for 24 h of cdc25A, CDK2, and cyclin A2 were assessed by western blot. Data were represented as mean ± SD, *n* = 3. ^*∗*^*P* < 0.05, compared to the control group.

The induction of cell cycle arrest is a vital characteristic of antitumor drugs that cause cell death and/or regulate tumor progression. Pterostilbene (0, 5, 10, and 20 *μ*M) and SCH772984 (0, 10 *μ*M) treatments for 24 h induced S-phase arrest in Jurkat and Hut-78 cells, respectively (Figures 2(a) and 2(c)). To explore the mechanism of pterostilbene-induced cell cycle arrest, cells were treated with pterostilbene (0, 5, and 10 *μ*M) for 24 h to test protein expression levels by western blot. cdc25A is an important mediator of the DNA damage checkpoint, so we further explored the complex network connecting cdc25A, CDK2, and cyclin A2 activity. As shown in Figure 2(e), cdc25A, CDK2, and cyclin A2 protein levels were dramatically decreased in the pterostilbene-treated group compared with the control group.

### 3.3. Pterostilbene and SCH772984 Induce Caspase-Dependent Apoptosis in T-Cell Leukemia/Lymphoma Cells

**Figure 3:**
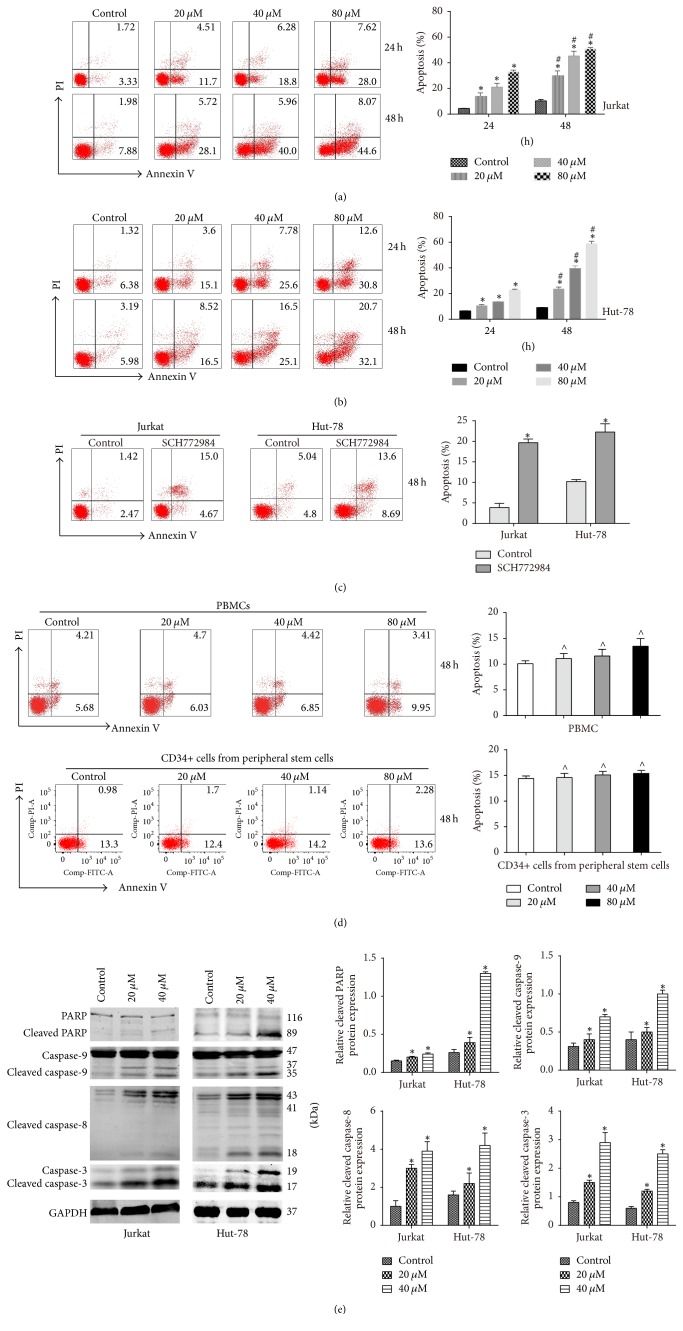
Pterostilbene and SCH772984 induce caspase-dependent apoptosis in T-cell leukemia/lymphoma cells. (a) Jurkat cells (3 × 10^5^ cells/mL) were treated with pterostilbene (0, 20, 40, and 80 *μ*M) and incubated for 24 h or 48 h. Data were shown as mean ± SD, *n* = 3. ^*∗*^*P* < 0.05, compared to the control group. ^#^*P* < 0.05, compared to the 24 h group. (b) Hut-78 cells (3 × 10^5^ cells/mL) were treated with pterostilbene (0, 20, 40, and 80 *μ*M) and incubated for 24 h or 48 h. Pterostilbene induced apoptosis of Jurkat and Hut-78 cells in a dose- and time-dependent manner. (c) Jurkat and Hut-78 cells were treated with SCH772984 10 *μ*M for 48 h. Data were shown as mean ± SD, *n* = 3. ^*∗*^*P* < 0.05, compared to the control group. (d) PBMCs and CD34+ cells from peripheral stem cells were treated with pterostilbene (0, 20, 40, and 80 *μ*M), and pterostilbene was of toxicity to normal cells. Data were shown as mean ± SD, *n* = 3. ^∧^*P* > 0.05, compared to the control group. (e) Protein levels treated with pterostilbene (0, 20, and 40 *μ*M) of cleaved caspase-3, cleaved caspase-8, caspase-9, and PARP for 48 h were detected by western blot.

From the cell cycle analysis, we found that pterostilbene and SCH772984 induced an increase in S-phase, which suggested that pterostilbene and SCH772984 might also induce apoptosis. To study pterostilbene- and SCH772984-induced cell death in Jurkat and Hut-78 cells, we performed an apoptosis assay by using the Annexin V-FITC/PI kit. The results showed that pterostilbene treatment for 24 h or 48 h markedly induced apoptosis of Jurkat (Figure 3(a)) and Hut-78 (Figure 3(b)) cells in a dose- and time-dependent manner. Compared with the group of control, SCH772984 (10 *μ*M) treatment increased the percentage of apoptotic cells in Jurkat and Hut-78 cells at 48 h (Figure 3(c)). Moreover, the result of Figure 3(d) showed that pterostilbene treatments (20, 40, and 80 *μ*M) have no toxicity in PBMCs and CD34+ cells from peripheral stem cells, further suggesting that pterostilbene is a safe agent for the treatment of T-cell leukemia/lymphoma. To further study the mechanism of apoptosis in pterostilbene-treated Jurkat and Hut-78 cells, we detected caspase-3, caspase-8, caspase-9, and PARP activities with pterostilbene treatment for 48 h by western blot. Our results demonstrated that pterostilbene treatment induced the cleavage of these proteins (Figure 3(e)).

### 3.4. Pterostilbene Induces Mitochondrial Membrane Potential Decline and Reactive Oxygen Species Generation in T-Cell Leukemia/Lymphoma Cells

**Figure 4:**
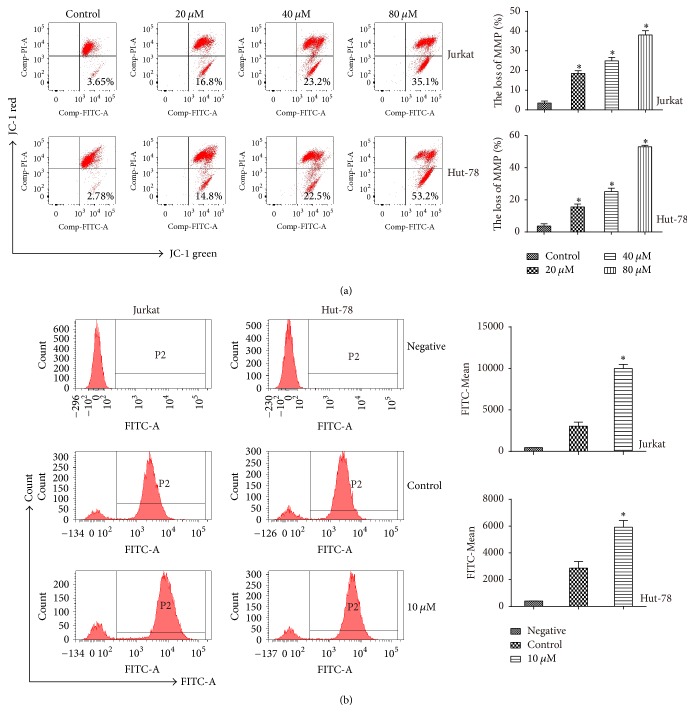
Pterostilbene induces MMP decline and ROS generation in T-cell leukemia/lymphoma cells. (a) Pterostilbene treatment (0, 20, 40, and 80 *μ*M) for 48 h induced mitochondrial depolarization in Jurkat and Hut-78 cells. Data were shown as mean ± SD, *n* = 3. ^*∗*^*P* < 0.05, compared to the control group. (b) Jurkat and Hut-78 cells treated with pterostilbene (0, 10 *μ*M) for 48 h by flow cytometry. In the negative group, Jurkat and Hut-78 cells were treated without pterostilbene and DCFH-DA. Data were shown as mean ± SD, *n* = 3. ^*∗*^*P* < 0.05, compared to the control group.

Mitochondrial membrane potential (MMP) is an important parameter of mitochondrial function that is used as an indicator of cell health. Therefore, we detected the effect of pterostilbene treatment for 48 h on MMP using the JC-1 MMP Detection Kit. The result showed that MMP was greatly decreased in pterostilbene-treated cells compared with the control group (Figure 4(a)). Recent studies have indicated that pterostilbene can increase reactive oxygen species (ROS) generation in cancer cells. To detect ROS levels in the DNA damage response, we evaluated ROS generation in Jurkat and Hut-78 cells treated with pterostilbene (0, 10 *μ*M) for 48 h by flow cytometry. In the negative group, Jurkat and Hut-78 cells were treated without pterostilbene and DCFH-DA. The results showed that the 10 *μ*M pterostilbene group had visibly increased ROS levels compared with the control group (Figure 4(b)).

### 3.5. ERK1/2 Phosphorylation Was Decreased following Pterostilbene Treatment

**Figure 5:**
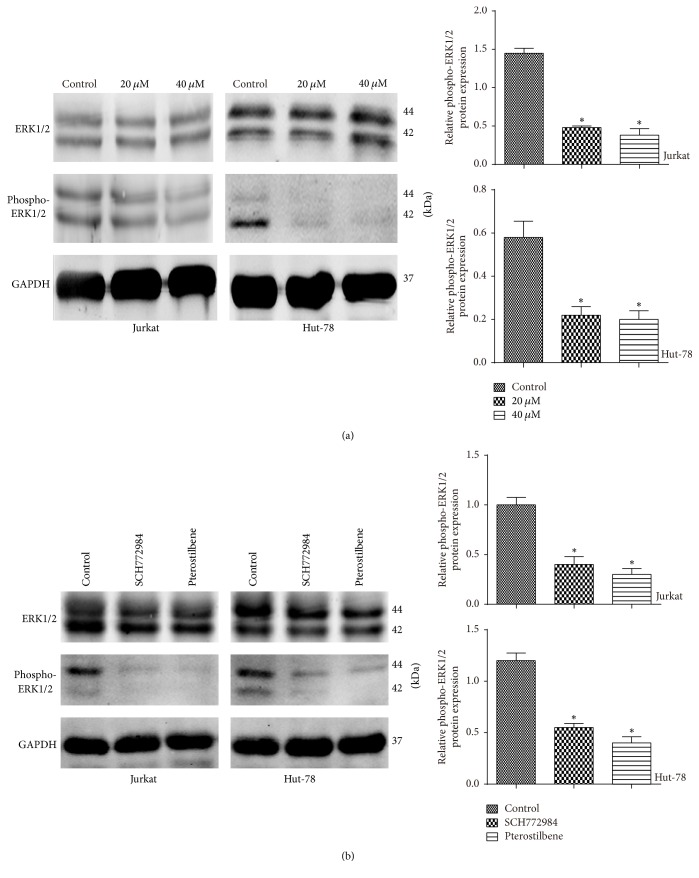
ERK1/2 phosphorylation was decreased following pterostilbene treatment for 48 h. (a) The expression levels of phospho-ERK1/2 and ERK1/2 were detected by western blot. Data were shown as mean ± SD, *n* = 3. ^*∗*^*P* < 0.05, compared to the control group. (b) Jurkat and Hut-78 cells were treated with SCH772984 (10 *μ*M) and pterostilbene (20 *μ*M) for 48 h, and the expression levels of phospho-ERK1/2 and ERK1/2 were detected by western blot. Data were shown as mean ± SD, *n* = 3. ^*∗*^*P* < 0.05, compared to the control group.

ERK1/2 is a member of the MAPK signaling pathways, and ERK1/2 activity in Jurkat and Hut-78 cells treated with pterostilbene (0, 20, and 40 *μ*M) for 48 h was assessed by western blot. As shown in Figure 5(a), cells treated with pterostilbene showed decreased levels of phospho (active)-ERK1/2, while there was no significant change in total ERK1/2. These data suggested that phospho-ERK1/2 suppression was induced by the apoptosis of T-cell leukemia/lymphoma cells following pterostilbene treatment. In addition, to further determine whether the ERK1/2 activity is mediated by pterostilbene, Jurkat and Hut-78 cells were treated with SCH772984 (10 *μ*M) and pterostilbene (20 *μ*M) for 48 h, respectively. We detected the expression level of ERK1/2 and phospho-ERK1/2 by western blot. As shown in Figure 5(b), SCH772984 decreased the level of the phospho-ERK1/2 as pterostilbene, further suggesting that pterostilbene could suppress the activity of ERK1/2.

## 4. Discussion

T-cell leukemia/lymphoma is one of the most aggressive hematological malignancies. Pterostilbene and resveratrol are phytoalexins that are found in plants and have various effects on mammalian cells. Recent studies have indicated that resveratrol is a powerful proapoptotic and antiproliferative agent for tumor cells in vitro and in vivo [17, 18]. As an analog of resveratrol, pterostilbene has known antitumor effects on cancer cells. Moreover, preclinical pterostilbene studies have shown that a variety of molecules and signaling pathways are involved in these antitumor effects. For example, pterostilbene induces apoptosis and autophagy in bladder cancer cells, while it was shown to inhibit tumor cell invasion in hepatoma HepG2 cells by decreasing MMP-9 activity [19, 20]. In our study, we showed that pterostilbene has dose-dependent cytotoxic effects on Jurkat and Hut-78 cells after 24 and 48 h treatment. This effect has also been observed in acute myeloid leukemia and the MOLT-4 human lymphoblastic leukemia cell line [21, 22]. At the same time, we found that the doses of pterostilbene we used in our present study are safe. Furthermore, we found that pterostilbene could decrease the growth of Jurkat and Hut-78 cells in a time-dependent manner. Flow cytometric analyses were consistent with these results, indicating that pterostilbene induced apoptosis in a dose- and time-dependent manner over a defined concentration range. Tumor cells are capable of endless proliferation, which is directly regulated by the cell cycle [23]. Cyclin-dependent kinases (CDKs) and cyclins play a key role in cell cycle progression, comprising the endogenous regulation and control of the process in all experimental models. Cyclin A2, CDK2, and cdc25A regulate the S-phase of the cell cycle, with cdc25A activating CDK2 as well as the cyclin-CDK complex. This process can be used as a marker of flux through the cell cycle, as high level cdc25A expression arises during rapid cellular growth [24, 25]. Thus, we detected the effect of pterostilbene on the cell cycle by flow cytometric analysis. The data showed that most cells treated with different concentrations of pterostilbene for 24 h were arrested in the S-phase. In addition, we investigated possible mechanisms that caused the S-phase arrest. Western blot analyses showed that pterostilbene treatment decreased cyclin A2, CDK2, and cdc25A levels. Apoptosis is a physiological process that is a normal part of growth and development. Therefore, we tested apoptosis rates in treated cells using the Annexin V-FITC/PI kit. The data showed that pterostilbene induced apoptosis of Jurkat and Hut-78 cells in a dose- and time-dependent manner. To further investigate the underlying mechanism, western blotting was used to assess apoptosis-related protein expression. The results showed that pterostilbene induced cleaved caspase-3, caspase-8, caspase-9, and PARP. Caspase-8 is the best characterized molecule of the extrinsic death pathways, and caspase-9 is part of an intrinsic pathway. Caspase-3 is the most significant member in the apoptotic pathway and can be activated by caspase-8 or caspase-9 [26]. To confirm activation of the intrinsic apoptosis pathway in pterostilbene-treated cells, we analyzed MMP by the JC-1 MMP Kit. The results demonstrated that pterostilbene induced mitochondrial depolarization in treated cancer cells. ROS is one of the cancerogenic factors due to its involvement in malignant transformation, but it also can be a killer of cancer cells [27]. Our data showed that pterostilbene induced apoptosis, possibly through the ROS generation pathway in Jurkat and Hut-78 cells. Therefore, pterostilbene-induced apoptosis may be regulated by the extrinsic and intrinsic apoptotic pathways in T-cell leukemia/lymphoma cells and was accompanied by caspase activation, which provoked programmed cell death. ERK1/2 belongs to the MAPK pathway, which plays important roles in tumorigenesis, proliferation, differentiation, and migration [27]. Recent studies have demonstrated that apoptosis is also associated with MAPK signaling [22]. Therefore, we further detected MAPK-related protein levels in pterostilbene-treated Jurkat and Hut-78 cells. The results showed that pterostilbene decreased phospho-ERK1/2 levels. Additionally, the effects of ERK inhibitor (SCH772984) on T-cell leukemia/lymphoma cells via Cell Counting Kit-8 and flow cytometric analysis were consistent with the findings of a recent study on pancreatic cancer [28], which indicated that ERK1/2 pathway may play an important role in pterostilbene-induced apoptosis and cell cycle arrest. In conclusion, our study showed that pterostilbene had antitumor activities, inducing apoptosis and S-phase cell cycle arrest, mainly through decreasing phospho-ERK1/2 levels. Together, these data demonstrate that pterostilbene might be a promising treatment for T-cell leukemia/lymphoma patients.
